# Effect of omega-3 supplementation versus placebo on acylation stimulating protein receptor gene expression in type 2 diabetics

**DOI:** 10.1186/2251-6581-13-1

**Published:** 2014-01-06

**Authors:** Payam Farahbakhsh-Farsi, Mahmoud Djalali, Fariba Koohdani, Ali Akbar Saboor-Yaraghi, Mohammad Reza Eshraghian, Mohammad Hassan Javanbakht, Maryam Chamari, Abolghassem Djazayery

**Affiliations:** 1 Cellular and Molecular Nutrition Department, School of Nutritional Sciences and Dietetics, Tehran University of Medical Sciences, Tehran, Iran; 2 Department of Nutrition and Biochemistry, School of Public Health, Tehran University of Medical Sciences, Tehran, Iran; 3 Community Nutrition Department, School of Nutritional Sciences and Dietetics, Tehran University of Medical Sciences, Tehran, Iran; 4 Department of Biostatistics, School of Public Health, Tehran University of Medical Sciences, Tehran, Iran

**Keywords:** Omega-3, Acylation stimulating protein receptor (C5L2), Type 2 diabetes mellitus, Gene expression

## Abstract

**Background:**

This randomized controlled trial investigated the role of omega-3 supplementation on C5L2 gene expression in type 2 diabetics.

**Methods:**

Subjects in the omega-3 group received 4 g omega-3 per day and subjects in the placebo group took four capsules of placebo per day for 10 weeks. Gene expression was measured by RT- PCR at the beginning and end of the study.

**Results:**

The results of this study show depletion in the omega-3 group, but the mean difference between two groups was not significant.

**Conclusions:**

Understanding the effect of the omega-3 pathway could contribute to targeting treatment of diabetes and its comorbidities.

## Background

Diabetes is a metabolic disorder that influences white adipose tissue (WAT) secretory adipokines such as leptin, adiponectin and acylation stimulating protein (ASP) [1]. ASP is an adipokine produced by adipose tissue that affects glucose metabolism and fat storage. ASP generally increases with obesity, type 2 diabetes, and cardiovascular disease [2]. In recent years, adipose tissue has been considered an endocrine organ responsible for the development of chronic diseases such as diabetes. In the two decades after the discovery of adipokines, the relationship between pancreatic cells and adipose tissue has been found to be a two-way pathway [3].

C5L2 is a protein with high chemical absorption that connects via separate sites to receptors such as C3a, C5a, C5a des-Arg, and C3a des-Arg (ASP). Triglyceride synthesis, however, occurs only when it connects to ASP [4]. C5L2 is an orphan G protein-coupled receptor (GPCR) family and has a high affinity to bind with ASP. It is a functional receptor for ASP and, if a signaling pathway exists via receptors, external agonists and antagonists can be clearly identified [5]. C5L2 knock-out mice show increased food intake, increased WAT, altered glucose/insulin metabolism, and change in adiponectin and insulin gene expression, which could lead to the development of insulin resistance. Disruption of C5L2 gene-induced macrophage presence in WAT contributes to obesity-associated disorders [6].

Factors that alter insulin resistance can induce changes in C5L2 gene expression [7]. Thiazolidinediones are anti-diabetic drugs with PPARγ agonistic characteristics [8]. Previous studies examined how thiazolidinediones such as rosiglitazone increase C5L2 mRNA and cell surface proteins [9]. Omega-3 fatty acids decrease blood triglyceride levels [10,11]. Regular intake of omega-3 may reduce the complications of diabetes [12,13]. The effect of pharmacological doses, such as anti-inflammatory effects, however, are not been clearly understood at the molecular level [11]. Recent studies show that PPARγ is a molecular target for omega-3 fatty acids which increases with n-3 fatty acids treatment [11,14].

It seems that omega-3 and thiazolidinediones use the same mechanism for PPARγ activation and the molecular effects of this agent have not been clearly explained. The present study examines the effect of omega-3 supplementation on C5L2 gene expression in type 2 diabetic patients.

## Methods

### Patients and supplementation

Forty-five type 2 diabetic mellitus (T2DM) patients (17 males, 28 females, 40–65 years of age; mean age: 53.77 yr) enrolled in this survey. One patient subsequently withdrew. Patients were informed of the goal and possible risks of the study and that they were free to withdraw at any time. This study was approved by the Tehran University of Medical Sciences (TUMS) ethical committee (ID: 15176). Informed consent forms were obtained from all participants after the purpose of the study was explained to them. This study registered on http://www.clinicaltrial.org as NCT01478776.

All patients were diagnosed by an endocrinologist based on fasting blood sugar. The exclusion criterion was having consumed omega-3 supplements within three months of the beginning of the study. No patient had complications of diabetes, thyroid disorder, nor did they use anti-obesity drugs. None were pregnant or breastfeeding. None was receiving thiazolidinediones or insulin therapy. All patients were requested to maintain their usual exercise and dietary habits. All participants were treated with metformin and glibenclamide.

All participants were divided into two randomly allocated groups (omega-3 or placebo) by random permuted blocks within the strata (BMI) method. The omega-3 group received 4 capsules of omega-3 (640 mg EPA, DHA, ALA, vitamin E) daily, and the placebo group took 4 placebo capsules per day for 10 wk. PBMC isolation, RNA extraction, cDNA synthesis, and real-time PCR for gene expression were done as described in previous studies [15,16]. Sequencing and information about primers are shown in Table 1.

**Table 1:** Primers sequencing and information

**Gene name**	**Sequence**	**Length**	**TM**	**CG%**
C5L2 forward	GCTGCAGTGTGTGGTGGACTAC	22	56.7	59.1%
C5L2 reverse	AAGAAACCGGATGGCAGTCA	20	56.6	50.0%
Gap DH forward	AAGGTGAAGGTCGGAGTCAAC	21	54.3	52.4%
Gap DH reverse	GGGGTCATTGATGGCAACAATA	22	58.0	45.5%

### Statistical analysis

Statistical analysis was done using SPSS 18.0 for windows. Data was expressed as mean ± SD. The Kolmogorov–Smirnov distribution test was used for departure from normality and nonparametric tests were used for data analyses that did not show normal distribution. Variables within and between groups were analyzed using the two-related sample test (Wilcoxon) and the two-independent sample test (Mann-Whitney U). If data had a normal distribution, the independent sample test and paired *t*-test were used for comparisons between groups, before and after treatment, and within groups, respectively. P-value < 0.05 was considered statistically significant.

## Results and discussion

### General information

A total of 44 patient volunteers (61.3% female, 38.6% male) were enrolled in this study. Anthropometrics of the treatment and placebo groups are shown in Table 2. There were no statistically significant differences in age, weight, height, BMI, waist and hip circumferences between the omega-3 and placebo groups (P = NS).

**Table 2:** Anthropometric data of patients and controls

	**Omega-3 group**		**Placebo group**	
	**Before**	**After**	**Before**	**After**
Age (years)	54.23 ± 1.64	-	53.32 ± 1.45	-
Weight (Kg)	69.21 ± 2.84	68.96 ± 2.91	63.57 ± 2.65	63.60 ± 2.78
Height (cm)	162 ± 2.11	-	156 ± 1.37	-
Waist circumflex (cm)	86.41 ± 2.33	86.15 ± 2.44	83.66 ± 2.10	83.16 ± 2.24
Hip circumflex (cm)	102.54 ± 1.62	101.83 ± 1.66	97.25 ± 1.74	97.22 ± 1.81
BMI (kg/m^2^)	26.19 ± 0.78	26.11 ± 0.84	25.93 ± 0.92	25.95 ± 0.98

All values are expressed as means ± SEM.

### C5L2 gene expression in PBMC extracted mRNA

**Figure 1:**
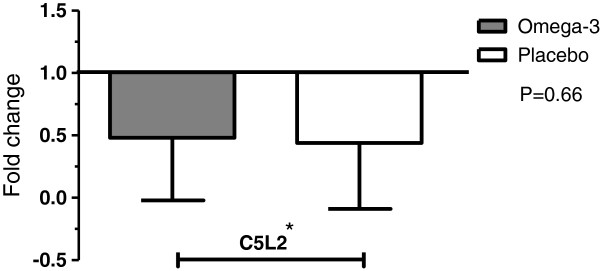
Gene expression of C5L2 in omega-3 and placebo groups.

The results showed that, by the end of the study, C5L2 gene expression as shown with cycle threshold modification between C5L2 and GAP DH, had decreased significantly in both the omega-3 and placebo supplemented groups (P = 0.01 and P = 0.001, respectively). Gene expression in the two groups were calculated with 2^-ΔΔct^, before and after treatment showed no significant difference (P = 0.66) (Table 3, Figure 1). The Prism software was used for explain fold changes modifications.

**Table 3:** ∆_CT and mean of C5L2 gene expression in PBMC

		**Omega-3 (n=22)**	**Placebo (n=22)**	**p valuea‡**
C5L2 gene expression in PBMC	Before	10.19 ± 2.87	10.90 ± 2.87	0.26
	After	9.16 ± 1.20	9.34 ± 1.07	0.77
	Difference	-1.03 ± 3.65	-1.55 ± 2.99	0.61
	p valueb*	0.01	0.001	
Mean of C5L2 gene expression in PBMC		0.48 ± 0.49	0.44 ± 0.53	0.66

Data are reported as means ±SD. ∆_CT = CT of target gene – CT of _GAP DH.

*two related sample tests (Wilcoxon) ‡ two independent sample tests (Mann Whitney U).

In this study, 44 diabetic patients enrolled in a 10 wk omega-3 and placebo supplementation study to determine the effect of omega-3 supplementation on C5L2. Fisette et al. have demonstrated that changes in ASP can influence diet-induced insulin resistance. Changes in the ASP-C5L2 pathway in obesity could accelerate obesity and co-morbidities such as T2DM and atherosclerosis [17]. Augmentation in ASP and TG in morbidly obese patients can alter the profile of the C5L2 receptor, ASP gene expression, and metabolic factors that show a compensatory response in patients [18]. Studies on knock-out mice show that C5L2 changes insulin expression and glucose metabolism and contributes to changes in insulin and adiponectin gene expression, and insulin resistance [6].

Insulin resistance and exercise change muscle function in obese diabetic men, contributing to changes C5L2 gene expression. These results indicate that insulin sensitivity may allow coupling of C5L2 levels to fat storage and utilization [7]. Acylation-stimulating protein and its receptor C5L2 contribute to adipocyte metabolism. In adiposities, sexual hormones such as progesterone and testosterone down-regulate C5L2. This is in contrast to thiazolidinediones such as rosiglitazone that up-regulate both C5L2 mRNA and C5L2 cell-surface protein [9]. ASP resistance is a product of sex-steroid hormones via C5L2 and may contribute to changes in the function of adipose tissue and insulin resistance in humans [19]. Tom et al. examined the effects of ASP on adipocytes and macrophages. Treatment with ASP on adipocytes had no effect on ligand binding of C5L2. Their results demonstrate ASP-induced inflammatory cytokines in adipocytes via PI3 kinase- and NFκB-dependent pathways, particularly at high physiologic doses [20].

In mature adipocytes treated with multiple doses of oleate or palmitate for 18 h, the C5L2 mRNA expression levels did not significantly decrease. These results suggest that down-regulation of C5L2 mRNA and protein may relate to impaired insulin [21]. Fusakio et al. [22] demonstrated that naive natural killer cells express C5aR and C5L2 mRNA, but did not express protein. Induced sepsis with Escherichia coli did not change C5L2 expression, but had a significant effect on IFN-γ and TNF-α serum levels. Zheng et al. [23] investigated C5L2 polymorphism in Chinese subjects and demonstrated that the 698CT genotype of C5L2 may be related to T2DM. The heterozygous expression of this gene may relate to metabolic abnormalities.

The demonstration of a functional ASP receptor using gain- and loss-of-function examinations is an important step in understanding the mechanisms of ASP function. Future studies of C5L2 activation and signaling, especially ligand binding, may help find C5L2 agonists-antagonists that might be modulators of the ASP pathway [5]. Although there is much left to learn about the role of C5L2 in humans, this investigation suggests that C5L2 may play a role in metabolic syndrome and diabetes.

## Conclusions

This study was a clinical trial of the effects of omega-3 supplementation on C5L2 gene expression in diabetic patients. It is postulated that omega-3 induced down-regulation of C5L2 gene expression in the omega-3 group.

## Abbreviations

ALA: A-Linolenic acid; ASP: Acylation stimulating protein; BMI: Body mass index; C5L2: C5a anaphylatoxin chemotactic receptor; CVD: Cardiovascular disease; DHA: Docosa hexaenoid acid; EPA: Eicosa pentaenoic acid; FPG: Fasting plasma glucose; GPCR: G protein-coupled receptor; Gap DH: Glyceraldehyde 3-phosphate de hydrogenase; INF-γ: Interferon gamma; PBMC: Peripheral blood mononuclear cell; PPAR-γ: Peroxisome proliferator-activated receptor γ; RCT: Randomized controlled trial; T2DM: Type 2 diabetes mellitus; TG: Tri acyl glycerol; TM: Temperature; TNF-α: Tumor necrosis factor alfa; TUMS: Tehran University of Medical Sciences; WC: Waist circumference; WAT: White adipose tissue.

## Competing interests

No potential conflicts of interest relevant to this article were reported.

## Authors’ contributions

PFF and MD; Design and conduct of the study, PFF, MR and MHJ; data collection, PFF and MRE; analysis, PFF, AASY, FK and SAD data interpretation and all authors have involved the manuscript writing. All authors read and approved the final manuscript.
